# Medical Journalists

**Published:** 1869-06-15

**Authors:** 


### *Medical Journalists.*

NEW ORLEANS, *May* 12, 1869.

EDITOR CHICAGO MEDICAL JOURNAL:

Your attention is particularly called to the enclosed copy of Proceedings of the Convention of Medical Journalists. Soliciting your co-operation, I am, with respect, Your obedient servant,

W. S. MITCHELL, M.D.,
*Permanent Sec'y.*

### MEETING AND ORGANIZATION.

On Wednesday evening, May 5th, a meeting of the Medical Society of Medical Journalists was held in the office, No. 1 Carondelet Street, New Orleans. The result of the assembly of prominent physicians was the formation of a permanent society; the election of N. S. Davis, M.D., President, and W. S. Mitchell, M. D., Permanent Secretary. The address of the Secretary is Lock Box 890, New Orleans. We append a report of the proceedings:

Pursuant to adjournment from the preliminary meeting on Tuesday, the meeting of Medical Journalists was called to order at 8 o'clock P. M., by Dr. N. S. Davis, of the Chicago *Medical Examiner*.

The Committee on Organization, through their chairman, Dr. Theophilus Parvin, of the *Western Journal of Medicine*, then presented the following preamble and plan of organization, which was unanimously adopted:

The Editors of Medical Journals in the United States, desiring to cultivate professional courtesies, to facilitate the conduct and general management of our journals, to promote their interests, their usefulness, and make them a still greater power for professional and popular good, and especially to advance the interests of medicine, hereby unite together under the following

ARTICLES OF ASSOCIATION.

*Name.*—The Association of American Editors.

*Purposes.*—The cultivation of friendly relations, mutual assistance, community of effort and means, where practicable, in a system of receiving foreign exchanges, and of sending our own journals abroad; in urging, with hearty concert, improvements in the present system of medical education, and a higher standard of preliminary eduction of those who desire to enter upon the study of medicine; the collection of vital statistics; the collecting of the names of all the regular physicians in the United States, age, place, and date of graduation, if a graduate; also, the same in reference to graduation at literary institutions, if such graduation has taken place.

*Meetings.*—These shall be held, commencing at 10 A. M., on the day preceding, and at the place of the annual meeting of the American Medical Association.

*Officers.*—President, Vice-President, Permanent Secretary, and Secretary.

The President, Vice-President, and Secretary shall be elected annually, and shall serve at the meeting of the succeeding year.

Committees shall be appointed where necessary for the carrying out of any of the special purposes of the Association.

These resolutions have been signed by the following delegates: Dr. N. S. Davis, Chicago Medical Examiner; Dr. Jas. M. Halloway, Richmond and Louisville Medical Journal; Dr. Wm. H. McPheeters, St. Louis Medical and Surgical Reporter; Dr. W. R. Bowling, Nashville Journal of Medicine; J. Berrien Lindsley, Nashville Journal of Medicine; Dr. Greeneville Dowell, Galveston Medical Journal; Dr. Samuel Logan, New Orleans Journal of Medicine; Dr. S. S. Herrick, New Orleans Journal of Medicine; Dr. E. W. Jenks and Dr. Geo. D. Andrews, Detroit Review of Medicine and Pharmacy; Dr. W. S. Mitchell, New Orleans Journal of Medicine, and Dr. S. M. Bemiss, New Orleans Journal of Medicine. The officers, as follows, were unanimously elected:

Dr. N. S. Davis, President; Dr. W. M. McPheeters, Vice President; Dr. W. S. Mitchell, Permanent Secretary, and Dr. J. Berrien Lindsley, Secretary.

The following resolutions were unanimously adopted:

That a Committee on Foreign Exchanges be appointed, to consist of Dr. Parvin, as chairman, and the Permanent Secretary.

That the Permanent Secretary be instructed to correspond with such regular medical journals of the United States as are not now represented, informing them of the objects of the organization, and inviting their co-operation.

That a Committee, consisting of Drs. Bowling, Dowell and Andrews, be appointed on the Registry of Physicians.

That Dr. Halloway be appointed a Committee on Revision.

That the President deliver, at the next meeting, an address on the history, progress, etc., of Medical Journalism in this country, and that the members of the Association furnish to him such material and information as they may be able to obtain.

That beside the members already signing the constitution, all physicians connected with regular medical journals, be considered members upon signifying, in writing, to the Permanent Secretary, their willingness to subscribe to the foregoing articles of agreement, until opportunity be afforded them of signing said articles.

That the President be requested to announce to the American Medical Association the formation and objects of this Association.

That these minutes be furnished to the secular papers, with a request that they be copied.

That Dr. Halloway be appointed a committee to arrange a general plan of commutation between medical journals.

That the Committee on Exchanges be instructed to arrange some general plan for the establishment of agencies in all the principal cities.

There being no further business, the meeting adjourned.

